# Curbing the lifestyle disease pandemic: making progress on an interdisciplinary research agenda for law and policy interventions

**DOI:** 10.1186/s12914-017-0131-5

**Published:** 2017-09-18

**Authors:** Brigit Toebes, Marlies Hesselman, Jitse P. van Dijk, Joost Herman

**Affiliations:** 1 Global Health Law Groningen Research Centre, Department of International Law, Faculty of Law, University of Groningen, Oude Kijk in ‘t Jatstraat 26, 9712 EK Groningen, the Netherlands; 2 Department of International Law, Faculty of Law, University of Groningen, Oude Kijk in ‘t Jatstraat 26, 9712 EK Groningen, the Netherlands; 3 Department of Community and Occupational Medicine, University Medical Center Groningen, University of Groningen, Ant. Deusinglaan 1, 9713 AV Groningen, the Netherlands; 4 Faculty of Arts, Oude Kijk in ‘t Jatstraat 26, 9712 EK Groningen, The Netherlands

**Keywords:** Noncommunicable diseases, Interdisciplinary research agenda, Law and policy

## Abstract

By 2030, noncommunicable diseases (NCDs) will be the leading cause of death in every region in the world. While law and policy have an important role to play in curbing this pandemic, our current understanding of how they can most effectively be used is still limited. This contribution identifies a number of gaps in current research and insists on an interdisciplinary research agenda between law, health science and international relations aimed at designing concrete proposals for laws and policies to curb the NCD pandemic, both globally and domestically.

## Background

In 2012, 38 million people died from noncommunicable diseases (NCDs), accounting for 68% of total deaths globally [1, 2]. NCDs include cardiovascular diseases, cancer, chronic respiratory diseases and diabetes. Importantly, NCDs are not merely a problem of high-income countries; their impact is universal. Around 28 million NCD-related deaths already occur in low and middle-income countries, and by 2030, NCDs will be the leading cause of death in every part of the world [2].

**Fig. 1:**
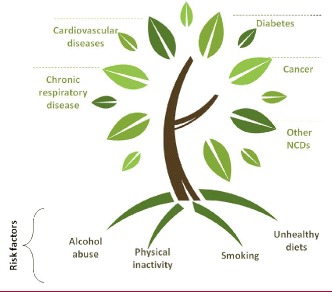
Modifiable Behavioural Risk Factors (Regional Office for the Eastern Mediterranean, WHO). Reprinted with permission from the WHO

The scale and universality of the problem make NCDs a pandemic phenomenon that requires a powerful international response. In this response, prevention is key, because much of the global NCD burden (40%) is linked to four “modifiable behavioral risk factors” that affect many countries: tobacco use, unhealthy diets, physical inactivity and harmful use of alcohol (Fig. 1; and per 2). Moreover, the rapid global spread of these risk factors is at least partially assisted by the globalization of the production, marketing and sales of harmful foods, beverages, alcohol, and tobacco products. Therefore, a strong, concerted international response is essential [3].

In recent years there has been a sizable international political commitment to curbing NCDs. In 2015, the United Nations General Assembly pledged in its Sustainable Development Goals to reduce NCDs by one-third by 2030. In parallel, the World Health Organization (WHO) adopted a set of nine voluntary targets on NCDs to be attained by 2025 [4]. This ‘2013–2020 WHO NCD Action Plan’ has been heralded as a paradigm shift for the response to NCDs, as it is the first international road map with a menu of policy options for States and other institutions to follow to achieve a substantial reduction in NCDs. Among the policy options mentioned are raising taxes on sugar, tobacco or alcohol, introducing packaging and labeling requirements, banning or limiting advertisements, and regulating the availability of products, or products’ ingredients [4].

Researchers from various disciplinary angles have engaged with NCDs and have made a meaningful contribution to the development of effective NCD laws and policies [5–8]. However, we observe that this type of research does not always receive the support and outreach it deserves. Specifically, many more efforts and resources should be allocated to interdisciplinary research endeavours, which, given the magnitude and complexity of the problem, deserve more attention.

While law and policy interventions have proven successful in curbing NCD incidence already, we need to learn more about the success factors that can be attributed to recent domestic and international instruments. To achieve this goal, we argue in favour of a truly integrated research agenda spanning health science, domestic and international legal scholarship, and international relations theory. In practice, this means that (traditional) law and policy-oriented NCD-research needs to build more on health science and international relations research, but also that health science can generate insights on the success of policy interventions. This is further explained below. In addition, domestic and international policy makers should better understand the important role of legal arrangements, while law-makers can learn from domestic and international policy realities and other challenges to which legal interventions need to respond.

The overall aim of our proposed integrated research agenda is to develop a comprehensive toolbox that can guide law and policy making, and assists identifying ‘good practice’ laws and policies for domestic governments to adopt [9]. This type of research needs to be developed in particular in Low and Middle Income Countries (LMICs), where little research on NCDs is carried out, in particular when it comes to evaluating good practice interventions in a local context [9].

## Linking health science with law and policy research

Domestically, there is increasing evidence that taxes on sugar sweetened beverages result in reduced consumption of these beverages, and that subsidies for fresh fruits and vegetables can increase fruit and vegetable consumption [10]. Moreover, bans on fast-food advertisements targeted at children have been linked to low childhood obesity rates in Canada [3]. Internationally, the Framework Convention on Tobacco Control represents a unique, binding treaty that has encouraged many countries to adjust their tobacco laws and policies [11].

At the same time, knowledge about the various regulatory options available to lawmakers and policymakers can be greatly improved [9]. Evidence so far suggests that modifiable risk factors can be addressed in a variety of ways, including targeting individual behavior, producers and sales points, each of which have specific advantages and disadvantages [12]. Laws can target consumers by creating incentives and disincentives that directly shape consumer behavior (e.g. taxes and subsidies), or by facilitating behavioral change (e.g. nutritional information). Yet, legislators can also regulate the industry or sales points, for example, by restricting unhealthy food promotion, restricting the sale of products (general bans, age-requirements), or by posing requirements on ingredients or packaging [13].

To identify effective law and policy options, in particular ‘best practices’, coordination between health science and law is crucial. Evidence of current patterns of NCDs, and interventions related to them, creates insights into which laws and policies can best be put into place. It also reveals how the implementation of laws and policies is succeeding. In respect of evidence, the estimate that in Europe obesity accounts for about 65–80% of new cases of Type 2 Diabetes is an important signal to legislators to reflect on the need to regulate risk factors such as unhealthy diets, which may be achieved by more effective regulation of certain foods [14]. At the same time, there is currently a lack of comprehensive data linking the incidence of NCDs to (certain types of) legal interventions. To design an adequate legal response, such data need to be specified per NCD. It is also important to generate data on the effects of adjusting modifiable determinants on health outcomes. For instance, knowledge about the health benefits of reduced salt intake (as a modifiable determinant) assists deciding on which legal and policy measures to take to help reduce the incidence of cardiovascular disease. Examples of measures to be taken in this area include setting appropriate standards for ingredients (for certain products), e.g. the levels of salt, requiring improved labeling, imposing taxes on certain products, and providing guidelines on cooking and salt intake. To choose the right policy option, a full investigation of the various specific legal and policy interventions that are possible, as well as those already undertaken in various domestic legal systems, needs to be carried out. Knowledge of these aspects is growing, but as yet incomplete [11, 12].

Despite overwhelming evidence of the scale of the NCD pandemic, and the importance and possibilities of curbing NCDs through prevention, we are aware that attempting to change lifestyle raises the difficult question as to whether and how national and international authorities *should* and *can* attempt to influence the behavioral and consumption patterns of individuals directly through regulation [15]. First, due to an emphasis on autonomy and personal responsibility, in particular in high- income countries, there is resistance in society to the implementation of such preventive measures [16].

Second, there is evidence that nudging, through regulation alone, may be insufficient because there are important underlying social determinants of health. For example, poverty, poor family relations, unemployment, and a lack of adequate education may increase engagement in certain risk factors [5, 6]. Successful policy interventions thus need to be embedded in multisectoral approaches spanning various policy sectors. From a research perspective, it requires a willingness and ability on the part of researchers to engage with scholars and insights from other disciplines [7].

Lastly, the practical and social role of companies should not be under-estimated. They heavily control essential aspects such as marketing, a product’s ingredients and presentation, and the availability of alternative products. These are important factors that influence people’s free choice which cannot be ignored. Moreover, while marketing bans or sales points restrictions may offer powerful tools to counter some of these forces, typically, tobacco, alcohol and food and beverages’ companies also have large budgets for lobbying and influencing markets and policy-makers in profound, if not subversive, ways. This type of influence may also have to be reigned in, nationally and internationally.

Despite the fact that NCDs pose the largest threat to future global health, they remain an under-emphasized area of inter-disciplinary research [8]. We argue that more research should be conducted aimed at filling the above-mentioned gaps and with the ultimate aim of identifying the best law and policy interventions [8]. To gain a full perspective, an interaction between the legal discipline and health science is key.

## Promoting social and legal change globally

Another under-researched dimension of NCDs concerns the question of how social and legal change can be brought about at both international and domestic levels. International relations’ research can give a crucial insight into the political processes required for new effective NCD laws and policies to be adopted. A close interaction between law and political science is required to study these mechanisms.

International laws and guidelines are essential for global agenda-setting in relation to NCDs and to ensure a comprehensive international response to the rising NCD pandemic. They present an important driving force for national authorities to prioritize certain health concerns and arrive at better regulation. So far the only legally binding international instrument that addresses a behavioral risk factor is the WHO’s Framework Convention on Tobacco Control (FCTC). This influential treaty, which was adopted in 2003 and is currently ratified by 179 countries, has led to a tightening of many domestic tobacco laws and to several domestic court cases addressing the harmful effects of tobacco [11]. There have been many calls over the last decade to adopt more instruments addressing the other risk factors [17]. The feasibility of new instruments should be explored further and important lessons regarding the form and content of such instruments can be drawn from the FCTC [18].

An important related question is whether new international standards should be pursued through treaty-making, or whether “soft-law” instruments in the form of Guidelines, Standards, Codes of Conduct or Action Plans can be equally effective in soliciting desired change and action. There is now evidence suggesting that more flexible, informal instruments might be equally, if not more effective in guiding States and other actor’s behavior, as long as the instrument is sufficiently precise and instructive, and has come about through a broad (multi-)stakeholder effort [19]. On the other hand, in terms of enforceability, binding international treaties can be very valuable instruments domestically, in domestic court proceedings and by legally requiring States to strengthen enforceable domestic laws. There is clearly a further research agenda here.

It is also important to understand how new international legal norms may emerge – or even can be actively pursued, framed, diffused, put into effect, and ultimately implemented. Models that explain ‘international norm dynamics’ in social constructivist international relations theory provide useful insights into how key domestic and international actors, including so-called “norm entrepreneurs”, interact with each other in relevant global networks and organizations such as the WHO. They explain how a sufficient momentum for the adoption of new instruments may come about, or not [20, 21]. In addition, such theories can explain the dynamics of “compliance”, “socialization” or “internalization” of norms, both nationally and internationally, which ultimately lead to law and policy action domestically [20, 22].

Finally, international relations theory suggests that any process of norm creation needs to start with agenda-setting and persuasion, or framing efforts by one or more dedicated “norm entrepreneurs” with (access to) sufficiently strong organizational platforms to spread their messages [20, 21]. One practical problem here is that the current international NCD law and policy agenda – and the NCD movement – is still nascent, and to some extent dispersed. There are at least four different NCDs that need to be addressed, and these are attributed to at least four complicated (and intersecting) risk factors. They are also covered by different disease-specific organizations (cancer foundations, diabetes foundations, etc.) [3]. A key question here may be to identify what leads to successful norm emergence, and how the efforts on various NCD agenda’s and related risk factors (e.g. alcohol, tobacco, sugar, salt) compare or may be combined (or not).

In pursuing effective global law and policy responses to NCDs, it will be crucial not only to understand health science and policy options on NCDs and risk factors, but also to understand the various critical factors that determine the success or failure of global health law-making efforts. Such critical factors can relate to the presence of successful norm entrepreneurs, or to understanding better how non-binding WHO instruments can inform and strengthen domestic responses to NCDs, in particular, in comparison to binding instruments such as the FCTC.

## Conclusions

The role of law and policy in regulating modifiable behavioral risk factors, including tobacco use, alcohol, unhealthy diets and low physical activity, has gained increased attention in international and domestic NCD debates. This article highlights that there is considerable evidence for successful international and domestic legal interventions, but also identifies salient knowledge gaps on the exact role of and options for suitable law and policy responses. Therefore, and in order to meet the ambitious goals on NCD reduction now set out in the Sustainable Development Goals, this article identifies key questions for a bold interdisciplinary research agenda which requires researchers in various fields to work more closely together.

## Abbreviations

FCTC: Framework Convention on Tobacco Control
NCDs: Noncommunicable diseases
WHO: World Health Organization
